# Selective depletion of radiolabeled HER2-specific antibody for contrast improvement during PET

**DOI:** 10.1080/19420862.2021.1976705

**Published:** 2021-09-30

**Authors:** Priyanka Khare, Wei Sun, Sreevidhya Ramakrishnan, Rafal Swiercz, Guiyang Hao, Su-Tang Lo, Kien Nham, Xiankai Sun, Raimund J. Ober, E. Sally Ward

**Affiliations:** a Department of Molecular and Cellular Medicine, Texas A&M University Health Science Center, College Station, Texas, USA; b Department of Biomedical Engineering, Texas A&M University, College Station, Texas, USA; c Cancer Sciences Unit, Centre for Cancer Immunology, Faculty of Medicine, University of Southampton, Southampton, UK; d Department of Radiology, University of Texas Southwestern Medical Center, Dallas, Texas, USA; e Department of Microbial Pathogenesis and Immunology, Texas A&M University Health Science Center, Bryan, Texas, USA

**Keywords:** Engineered Fc fusions, PET, HER2

## Abstract

The prolonged in vivo persistence of antibodies results in high background and poor contrast during their use as molecular imaging agents for positron emission tomography (PET). We have recently described a class of engineered Fc fusion proteins that selectively deplete antigen-specific antibodies without affecting the levels of antibodies of other specificities. Here, we demonstrate that these Fc fusions (called Seldegs, for selective degradation) can be used to clear circulating, radiolabeled HER2-specific antibody during diagnostic imaging of HER2-positive tumors in mice. The analyses show that Seldegs have considerable promise for the reduction of whole-body exposure to radiolabel and improvement of contrast during PET.

## Introduction

The use of engineering approaches to generate antibodies of high affinity and specificity for target has led to an expansion of interest in using these agents as diagnostic and theranostic imaging agents. However, antibodies of the IgG class are endowed with long *in vivo* persistence due to their ability to bind to the neonatal Fc receptor (FcRn).^1^ This longevity results in high background, poor contrast and, if radiolabeled for detection using positron emission tomography (PET), potential radiation damage to normal tissue.^2–4^ The current study is directed toward overcoming these limitations by using a novel approach to selectively clear target-specific antibody that is not tumor-bound during PET.

We recently developed engineered antigen-Fc fusion proteins that selectively clear antigen (target)-specific antibodies without affecting the levels of endogenous antibodies of other specificities.^5^ These engineered fusion proteins, named Seldegs (for *sel*ective *deg*radation), comprise an antigen component that is fused to an Fc-based targeting component. The targeting component is engineered to bind to the internalizing receptor, FcRn, with substantially increased affinity at near neutral and acidic pH, whereas the antigen component captures antigen-specific antibodies.^5,6^ The antibodies that bind to the antigen component are bivalent, and therefore to minimize the formation of multivalent immune complexes, Seldegs are engineered with knobs-into-holes mutations^7^ to display the antigen as a monomer. The Fc is also engineered with mutations to substantially reduce or ablate interactions with FcγRs or complement.^8^ The high affinity binding of Seldegs to FcRn leads to the rapid internalization of antibody-Seldeg complexes into FcRn-expressing cells followed by their lysosomal degradation.^5^ Consequently, following injection into mice, Seldeg delivery results in the selective depletion of antigen-specific antibodies.^5^

Our recent studies have demonstrated that specifically designed Seldegs can be used to deplete antibodies specific for the autoantigen, myelin oligodendrocyte glycoprotein (MOG), and the tumor target human epidermal growth factor receptor-2 (HER2).^5,9^ In addition, we have shown that the depletion of MOG-specific antibodies results in the amelioration of antibody-mediated disease in mice.^9^ However, the efficacy of Seldegs in reducing background and enhancing contrast during PET analyses in tumor-bearing mice has not been investigated. Here, we describe a strategy using a Seldeg that targets HER2-specific antibodies such as pertuzumab^10^ to substantially improve contrast during PET.

## Results

**Figure 1.**
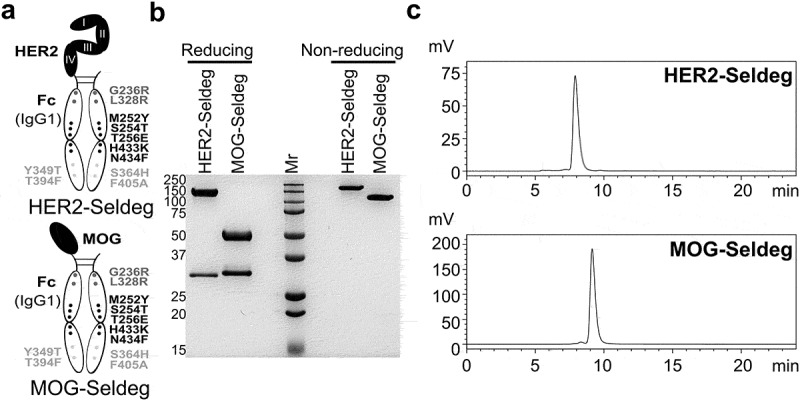
Design and size exclusion analyses of Seldegs. (a) Schematic representation of the design of the Fc fusion proteins. HER2-Seldeg and MOG-Seldeg consist of domains I–IV of HER2 and the extracellular domain of MOG, respectively, fused to a heterodimeric, human IgG1-derived Fc.^5^ The mutations to ablate FcγR binding,^8^ enhance FcRn binding^11^ and drive heterodimer formation (knobs-into-holes mutations^7^) are shown. Analyses of the purified Seldegs using SDS-PAGE (run under reducing and non-reducing conditions) (b) and a Phenomenex Yarra 3 µm SEC-3000 column (Phenomenex, 00 H-4513-K0) (c). For (b), the sizes of molecular weight (Mr) standards are shown in kDa on the left margin

The design of Seldegs and their analysis following purification are shown in Figure 1. For use as a control for the Seldeg targeting HER2-specific antibodies (HER2-Seldeg), a Seldeg in which extracellular domains I–IV of HER2 were replaced with the extracellular domain of MOG (MOG-Seldeg) was produced (Figure 1a). SDS-PAGE and HPLC analyses indicated that HER2-Seldeg and MOG-Seldeg were heterodimeric (Figure 1b, non-reducing conditions) and free of aggregates (Figure 1c). The interactions of HER2-Seldeg with mouse FcRn and the HER2-specific antibody, pertuzumab,^10^ were characterized using surface plasmon resonance (SPR; BIAcore) and resulted in equilibrium dissociation constants (K_D_s) at pH 7.4 of 15.6 nM and 17.5 nM for mouse FcRn and pertuzumab, respectively (Figure 1a,Figure 1b). In addition, the K_D_ of the interaction of HER2-Seldeg with mouse FcRn at pH 6.0 was 5.1 nM (Figure 1c). This behavior is consistent with earlier studies demonstrating that the FcRn-enhancing mutations (‘MST-HN’) that are present in the Fc of Seldegs lead to substantial increases in binding to mouse FcRn.^5,11^ In earlier studies, both HER2- and MOG-Seldegs have been shown to deplete antigen-specific antibodies, whilst not affecting the levels of endogenous IgG, in mice.^5^

**Figure 2.**
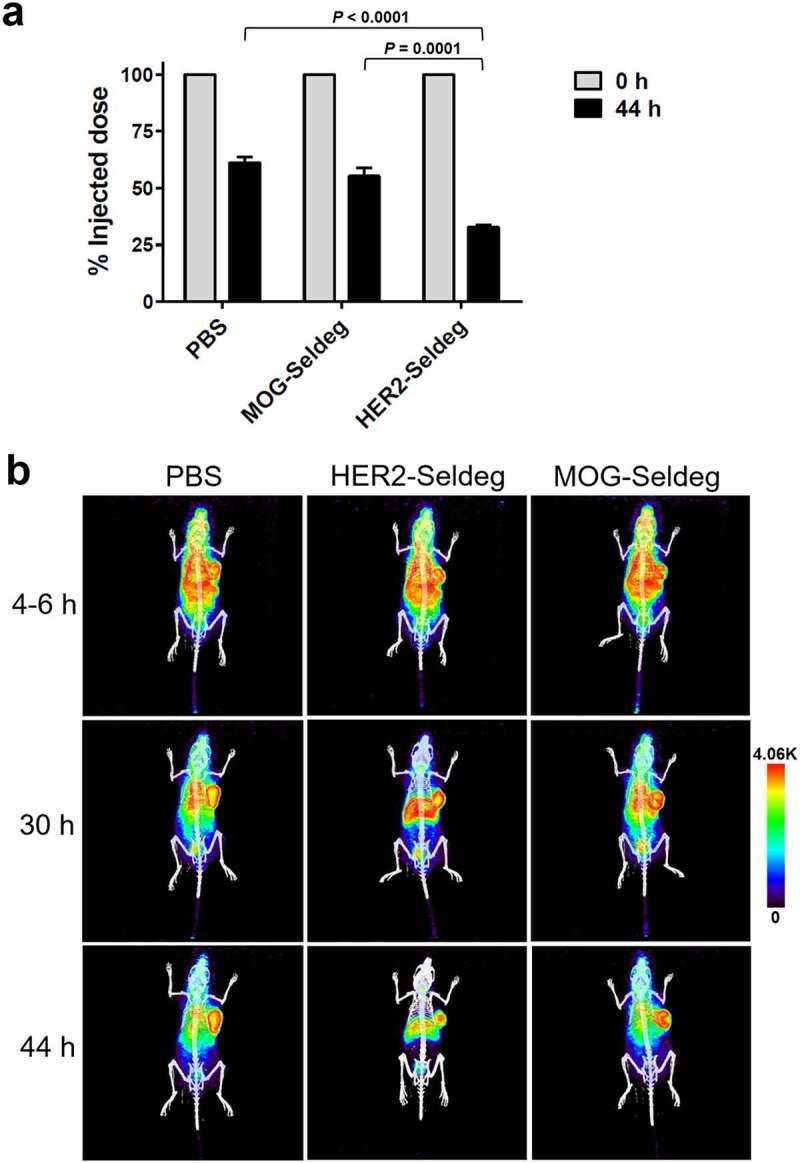
Effect of delivery of HER2-Seldeg following injection of radiolabeled pertuzumab into tumor-bearing mice. Twenty-four hours following intravenous injection of ^124^I-pertuzumab, mice (n = 3 mice/group) were intravenously injected with 51 µg of HER2-Seldeg, 31 µg of MOG-Seldeg or PBS vehicle. Seldeg amounts were equimolar with the amount of injected pertuzumab. (a) Whole body counts immediately following radiolabeled pertuzumab injection and 44 h later (20 h post-Seldeg delivery) are shown, with data normalized to the injected dose. Error bars indicate SD and *p* values for significant differences for HER2-Seldeg treatment groups vs. control groups are shown (one-way ANOVA and Tukey’s multiple comparison test). (b) PET/CT images acquired at 4–6, 30, and 44 h after injection of radiolabeled pertuzumab. The time points correspond to 18–20 h prior to, and 6 and 20 h following, delivery of Seldeg, respectively. Data for one representative mouse per treatment group is shown, with linear scale bars. Data shown are representative of two independent experiments (n = 3 mice per treatment group in each experiment)

**Figure 3.**
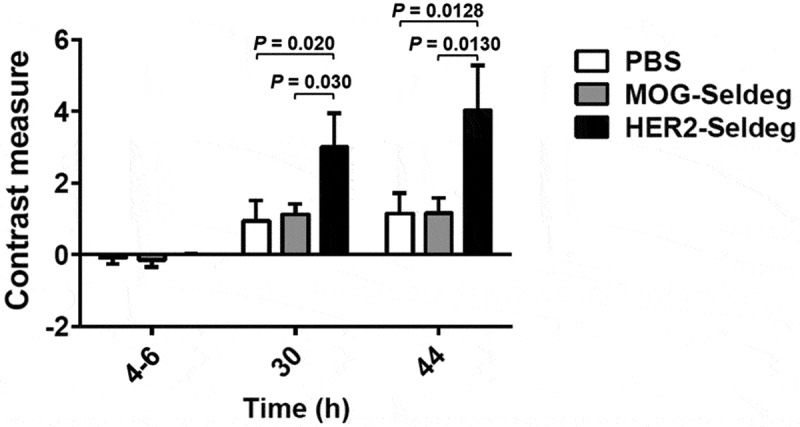
Effect of delivery of Seldeg on PET contrast. Tumor-bearing mice were treated as in Figure 2. Contrast measures were determined at 4–6, 30, and 44 h after injection of radiolabeled pertuzumab. These time points correspond to 18–20 h prior to, and 6 and 20 h following, Seldeg delivery, respectively. Error bars indicate SD and *p* values for significant differences for HER2-Seldeg treatment groups vs. control groups are shown (one-way ANOVA and Tukey’s multiple comparison test). Data shown are representative of two independent experiments (n = 3 mice per treatment group in each experiment)

To analyze the ability of the HER2-Seldeg to improve contrast during diagnostic imaging, mice were implanted with the HER2-overexpressing tumor cell line, HCC1954. Following 7 days, tumor-bearing mice were injected with ^124^I-labeled pertuzumab. The mice were imaged using PET at 4–6 h post-injection. HER2-Seldeg or controls (MOG-Seldeg or PBS vehicle) were delivered in mice (n = 3 mice per treatment group) at 24 h post-pertuzumab injection. Delivery of the HER2-Seldeg resulted in a substantial decrease in whole body levels of radiolabel compared with that in control groups at 20 h post-Seldeg delivery (Figure 2a). Mice were imaged using PET at 6 or 20 h post-Seldeg injection (Figure 2b; Supplementary Videos 1–6). The delivery of HER2-Seldeg led to 2.5-3-fold higher contrast for the radiolabeled pertuzumab compared with that in control groups (Figure 3; Figure 2). In addition, and consistent with our earlier biodistribution studies,^9^ Seldeg injection resulted in the delivery of targeted antibody to the liver (Figure 2b). In an additional experiment, we observed that, although imaging mice at 6 or 8 h post-Seldeg delivery led to similar contrast measures, analyses at 3 h resulted in ~2-fold lower contrast (n = 4 mice, treated with HER2-Seldeg), indicating that by comparison with 6–8 h (Figure 3), Seldeg treatment for 3 h is suboptimal for contrast improvement.

## Discussion

In this study we demonstrated that engineered antigen-Fc fusions with the ability to selectively degrade antigen-specific antibodies can be used to improve contrast during the use of radiolabeled HER2-specific antibody, pertuzumab, to image HER2-positive tumors. This novel approach is expected to be broadly applicable, using appropriately engineered antigen-Fc fusions, to imaging with antibodies or other molecular agents specific for different (tumor) targets. Importantly, through their ability to bind and internalize antibodies with a particular specificity into lysosomes in cells, Seldegs do not affect the levels of endogenous, protective antibodies.^5^

When used as imaging agents, the prolonged *in vivo* persistence of antibodies necessitates intervals of up to 7 days between the delivery of radiolabeled antibody and PET (or other imaging modality).^12,13^ This wait time can lead to undesirable exposure of normal tissue to radiation. Pretargeting, involving the delivery of unlabeled, derivatized antibody followed by injection of radiolabeled PET probe that is designed to conjugate to the antibody, has been developed as an approach to overcome this problem.^14,15^ Shortcomings of pretargeting are the need for wait times for unbound, long-lived antibody to clear and the use of high levels of radiolabel. The application of Seldegs is expected to substantially shorten the exposure time to radiolabel due to the ability of these Fc fusions to deplete targeted antibody levels in the circulation within several hours of delivery.^5^ In this context, although the use of antibody fragments with considerably reduced *in vivo* half-lives can be used to reduce radiation exposure times,^16,17^ the short half-life of the imaging agent can limit the levels that accumulate in the tumor. Alternatively, high dose intravenous immunoglobulin (IVIG), which acts as a competitor with endogenous IgG for binding to the salvage receptor, FcRn, has been used to clear background during imaging with labeled antibodies.^3,12^ In addition to reducing radiolabeled antibody levels, this can result in decreases in endogenous antibody (IgG) levels for up to several weeks due to the pharmacokinetic behavior of IVIG.^18,19^ Alternatively, an engineered, high affinity antagonist of FcRn with relatively short *in vivo* persistence has been used to enhance PET contrast,^20,21^ but this approach also results in the lowering of IgGs independent of their specificity.

In summary, Seldegs have the potential to provide an alternative, highly specific strategy to improve contrast and reduce background during diagnostic imaging. Further, the utility of Seldegs not only has relevance to the effective use of antibodies in imaging, but also to other classes of molecular diagnostics for which the regulated clearance of circulating agent is desirable.

## Materials and methods

### Antibodies and radiolabeling

Pertuzumab^10^ was obtained from the UT Southwestern Medical Center Pharmacy (University of Texas Southwestern Medical Center, Dallas, TX) and was labeled with ^124^I (PerkinElmer) using the Iodogen reagent in pre-coated tubes (Pierce).^11^ The Na^124^I (369 µCi; 13.65 MBq; PerkinElmer) solution was firstly mixed with Na^nat^I (667 pmol, 0.1 µg, 10 µl) solution and then added to each Iodogen tube containing ~246 µg pertuzumab in phosphate-buffered saline (PBS). The iodination reaction was carried out for 15 min at room temperature. At the end of the reaction, the product solution was mixed with 10 µl of 10 mg/ml L-ascorbic acid solution in a 5 ml tube to reduce radiolysis of the radiolabeled antibody,^22^ and radiolabeled pertuzumab was isolated using a 10 kDa Amicon Ultra-4 centrifugal filter unit (Millipore Sigma). Radiolabeled pertuzumab was analyzed using instant thin layer chromatography medium and PBS as the developing solution, and the radiochemical purity of the ^124^I-labeled pertuzumab was over 95%.

### Expression and purification of Seldegs

Expression constructs encoding heterodimeric HER2-Seldeg or MOG-Seldeg^5^ were transiently transfected into HEK-293 F cells (Life Technologies) using Gibco Expi293 expression system kits (Life Technologies). These heterodimeric constructs contain the following mutations in the two constituent (human IgG1-derived) Fcs: FcRn-enhancing (MST-HN = M252Y/S254T/T256E/H433K/N434F^11^) and FcγR/complement C1q ablating mutations (G236R/L328R^8^). The Fc and antigen-Fc also contain knobs-into-holes mutations (S364H/F405A and Y349T/T394F,^7^ respectively) to drive heterodimer formation. Recombinant proteins were purified from culture supernatants using protein A-Sepharose followed by size exclusion chromatography (Hiload 16/600 Superdex 200 gel filtration column; GE Healthcare) in yields of ~17 mg/liter culture (HER2-Seldeg) and ~133 mg/liter culture (MOG-Seldeg). Purified proteins were analyzed using a Phenomenex Yarra 3 µm SEC-3000 column (Phenomenex, 00 H-4513-K0).

### Binding analyses

SPR experiments were performed using a BIAcore T200 (GE Healthcare). To analyze the interactions of HER2-Seldeg with mouse FcRn and pertuzumab (clinical grade), recombinant mouse FcRn (generously provided by Dr. Richard Stopforth, University of Southampton) or pertuzumab were immobilized on flow cells of a CM5 chip using amine coupling chemistry. The coupling densities were 672.8 RU (mouse FcRn) and 572.6 RU (pertuzumab). HER2-Seldeg was injected (350 μl/injection) at different concentrations in duplicate or triplicate injections at 25°C using programmed methods at a flow rate of 10 μl/min in DPBS (Lonza; pH 7.4 or adjusted to pH 6.0) plus 0.1% v/v Tween 20. Flow cells were regenerated at the end of each cycle using 0.1 M NaHCO_3_/0.15 M NaCl pH 9 (mouse FcRn) or 0.1 M glycine/1 M NaCl/10% v/v glycerol pH 2.0 (pertuzumab). Equilibrium dissociation constants (K_D_s) were estimated using custom-written software and a 1:1 interaction model,^23^ leading to apparent K_D_s for the mouse FcRn:Seldeg interactions which are bivalent.

### Mice and tumor implantation

All animal experiments described in this study were approved by the Texas A&M University and UT Southwestern Medical Center Institutional Animal Care and Use Committees. Experiments were carried out in 8–10-week-old SCID BALB/c female mice (Jackson Laboratory) that were bred in a pathogen-free facility at Texas A&M University. To implant tumor xenografts, HCC1954 cells (0.5 x 10^6^ cells/mouse) suspended in 0.1 mL of RPMI-1640/Matrigel (Corning Inc.) (1:1 ratio of medium:Matrigel) were injected into mammary fat pad number 3 of each mouse using a 22-gauge needle as described previously.^20^ Tumor-implanted mice were shipped to UT Southwestern Medical Center for use in PET experiments.

### Seldeg treatment in mice

Seven days following tumor implantation, when tumors were approximately ~175-300 mm^3^ in volume, mice were divided into groups (n = 3 mice per treatment group) and injected intravenously with ^124^I-labeled pertuzumab. Thyroid uptake of radiolabeled iodine was reduced by adding Lugol solution to drinking water 48 h before the injection of radiolabeled proteins. To block stomach uptake of radiolabeled iodine, mice underwent gastric lavage using 1.5 mg potassium perchlorate in 0.2 ml PBS, 30 min before injection of radiolabeled (^124^I-labeled) antibody (2.96–3.33 MBq, 60 µg antibody/mouse in a volume of 100 μl via tail vein injection). Mice were injected intravenously with 51 μg HER2-Seldeg, 31 μg MOG-Seldeg, or vehicle (PBS), 24 h after injection of radiolabeled pertuzumab. These Seldeg doses are equimolar with the injected dose of pertuzumab. Computed tomography (CT) and PET images were acquired at the indicated times.

### Image acquisition

Imaging of mice was performed using a Siemens Inveon PET-CT Multimodality System (Siemens Medical Solutions, Inc., Knoxville, TN) with an intrinsic spatial resolution of 1.5 mm for PET. The CT images were acquired with 180 projections over a full 360 degree rotation at a voltage of 80 kV, current of 500 µA, exposure time of 140 ms per projection, and binning factor of 4. The CT images were reconstructed using COBRA reconstruction software (Exxim Computing Corp.) with a downsampling factor of 2. The reconstruction profile for CT was set to interpolate bilinearly using a Shepp-Logan filter. PET was carried out immediately following CT data acquisition, and PET images were acquired for 20 min with the acquisition energy window set to 350–650 keV for ^124^I. PET images were reconstructed with a 3D Ordered-Subset Expectation Maximization followed by Maximum a Posteriori (OSEM3D/MAP) algorithm using the Siemens Inveon Acquisition Workplace (Siemens Medical Solutions, Inc.).

### Image analysis

PET and CT images were registered in the AMIDE software package.^24^ Linear intensity adjustments with a zero-minimum threshold were applied to display the PET images. The maximum thresholds were adjusted equally across all three groups and time points. For measuring the contrast, two ellipsoidal regions of interest (ROIs) were defined manually. One ROI encompassed the tumor in all planes. A second ROI was defined in the thorax area to measure the background intensity. The mean background intensity and the mean tumor intensity were calculated by taking the average of the intensity of the voxels within both the ROIs. This was carried out for each mouse. The dimensionless Contrast Measure (CM) is defined as:

$$CM=(I_{T}-I_{B})/I_{B}$$

where I_T_ and I_B_ denote the mean tumor intensity and the mean background intensity, respectively. 3D animal images were generated using the 3D volume rendering tool available in the AMIDE software.

### Statistical analyses

Tests for statistical significance between treatment groups were performed using one-way analysis of variance (ANOVA) with Tukey’s multiple comparison test and plotted in GraphPad Prism, V6.0 (GraphPad Software). *p* values less than 0.05 were considered to be significant.

## Supplementary Material

Supplemental Material

## Data Availability

The data that supports the findings in this study are available upon request from the corresponding authors.

## Abbreviations

CT, computed tomography

HER2, human epidermal growth factor receptor 2

IVIG, intravenous immunoglobulin

MOG, myelin oligodendrocyte glycoprotein

PET, positron emission tomography

## Code availability

Software used to analyze the PET data is available upon request.
