# Supplementary material for: Selective depletion of radiolabeled HER2-specific antibody for contrast improvement during PET
Source: MAbs. 2021 Sep 30;13(1):1976705. doi: 10.1080/19420862.2021.1976705 (PMC8489906; doi:10.1080/19420862.2021.1976705)
Supplement: Supplemental Material [file KMAB_A_1976705_SM4837.zip › Supplementary information/supplementary data_082021_clean.docx]

**Supplementary data**

**Selective depletion of radiolabeled HER2-specific antibody for PET contrast improvement**

Priyanka Khare, Wei Sun, Sreevidhya Ramakrishnan, Rafal Swiercz,Guiyang Hao, Su-Tang Lo, Kien Nham, Xiankai Sun, Raimund J. Ober and E. Sally Ward

**
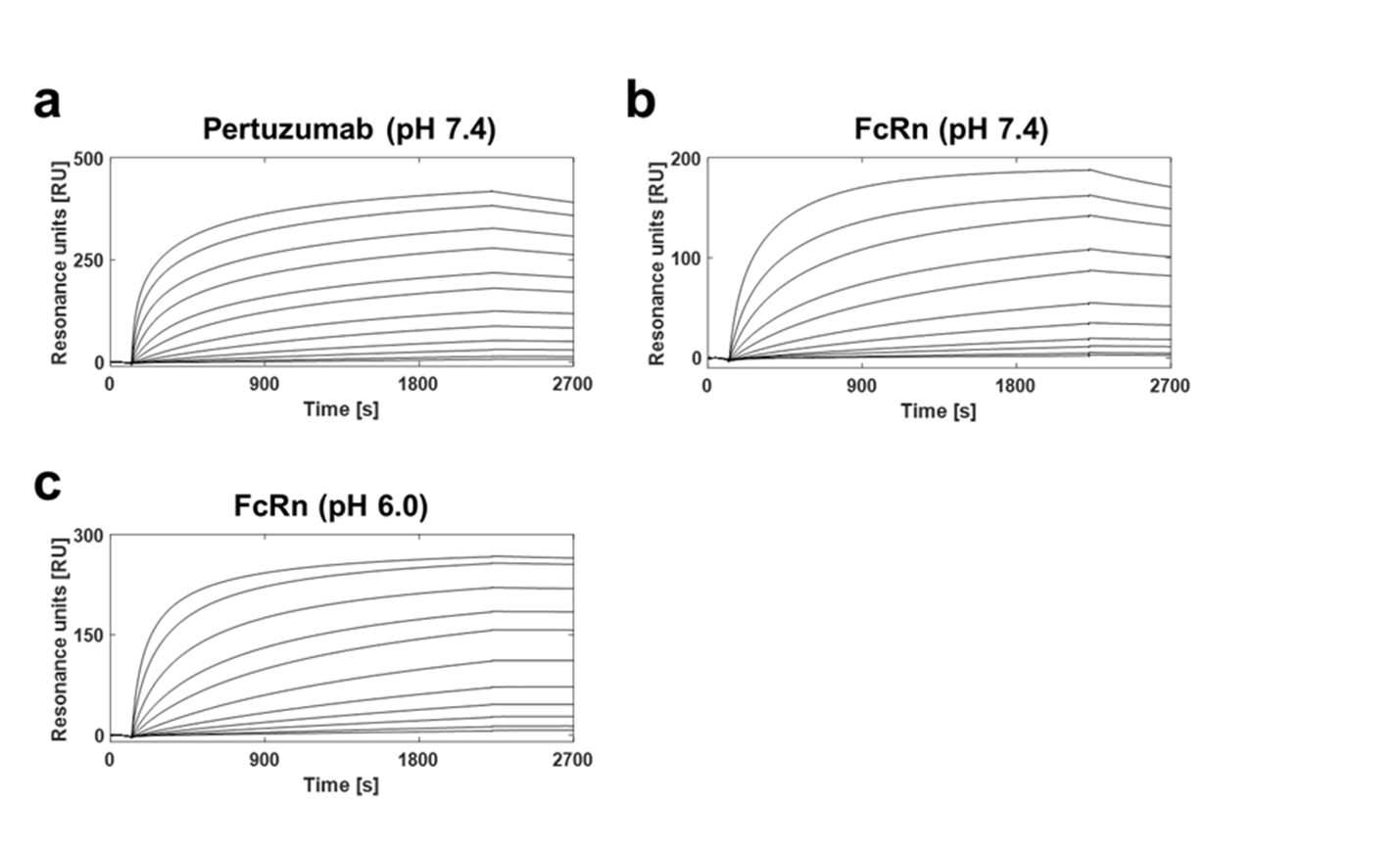
**

**Supplementary Figure 1.** Surface plasmon resonance analyses of the interaction of immobilized pertuzumab (572.6 RU) or mouse FcRn (672.8 RU) with HER2-Seldeg. HER2-Seldeg was injected at either pH 7.4 (a,b) or 6.0 (c) at concentrations ranging from 0.195-400 nM (a), 0.195-200 nM (b) or 0.195-100 nM (c) at a flow rate of 10 μl/min and equilibrium dissociation constants (K_D_s) determined. Flow cells were regenerated at the end of each cycle using 0.1 M glycine/1 M NaCl/10% v/v glycerol pH 2.0 (pertuzumab) or 0.1 M NaHCO_3_/0.15 M NaCl pH 9 (mouse FcRn). Representative traces for duplicate or triplicate injections for each analyte concentration are presented.


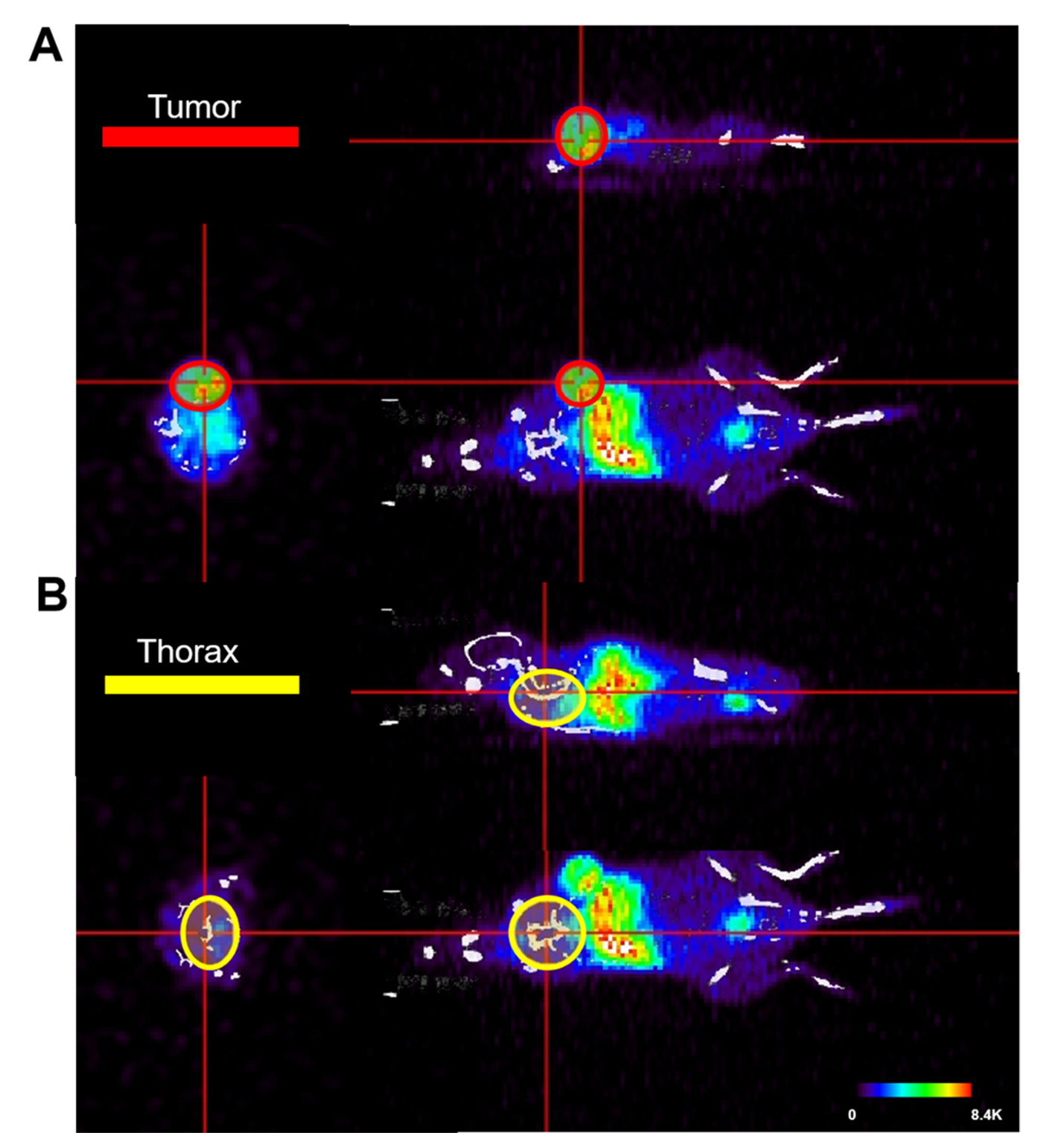


**Supplementary Figure 2.** Different views of the regions of interest (ROIs). The tumor ROI (a) and the thorax ROI (b) were overlaid with PET and CT images to determine contrast measures (ROIs for a PBS-treated mouse from the experiment described in Figure 2 are shown as an example). The intensity in the displays is linearly adjusted.

**Supplementary Videos**

**Supplementary Video SV1:** PET/CT 3D volume rendering of the PBS-treated mouse shown in Figure 2b at 30 h following ^124^I-pertuzumab injection (6 h post-PBS injection).

**Supplementary Video SV2:** PET/CT 3D volume rendering of the HER2-Seldeg-treated mouse shown in Figure 2b at 30 h following ^124^I-pertuzumab injection (6 h post-Seldeg injection).

**Supplementary Video SV3:** PET/CT 3D volume rendering of the MOG-Seldeg-treated mouse shown in Figure 2b at 30 h following ^124^I-pertuzumab injection (6 h post-Seldeg injection).

**Supplementary Video SV4:** PET/CT 3D volume rendering of the PBS-treated mouse shown in Figure 2b at 44 h following ^124^I-pertuzumab injection (20 h post-PBS injection).

**Supplementary Video SV5:** PET/CT 3D volume rendering of the HER2-Seldeg-treated mouse shown in Figure 2b at 44 h following ^124^I-pertuzumab injection (20 h post-Seldeg injection).

**Supplementary Video SV6:** PET/CT 3D volume rendering of the MOG-Seldeg-treated mouse shown in Figure 2b at 44 h following ^124^I-pertuzumab injection (20 h post-Seldeg injection).
